# A memetic optimization algorithm for multi-constrained multicast routing in *ad hoc* networks

**DOI:** 10.1371/journal.pone.0193142

**Published:** 2018-03-06

**Authors:** Rahab M. Ramadan, Safa M. Gasser, Mohamed S. El-Mahallawy, Karim Hammad, Ahmed M. El Bakly

**Affiliations:** 1 Department of Basic and Applied Sciences, Arab Academy for Science, Technology and Maritime Transport, Cairo, Egypt; 2 Department of Electronics and Communications, Arab Academy for Science, Technology and Maritime Transport, Cairo, Egypt; Northeast Normal University, CHINA

## Abstract

A mobile ad hoc network is a conventional self-configuring network where the routing optimization problem—subject to various Quality-of-Service (QoS) constraints—represents a major challenge. Unlike previously proposed solutions, in this paper, we propose a memetic algorithm (MA) employing an adaptive mutation parameter, to solve the multicast routing problem with higher search ability and computational efficiency. The proposed algorithm utilizes an updated scheme, based on statistical analysis, to estimate the best values for all MA parameters and enhance MA performance. The numerical results show that the proposed MA improved the delay and jitter of the network, while reducing computational complexity as compared to existing algorithms.

## Introduction

A Mobile *Ad hoc* NETwork (MANET) is a collection of arbitrarily located nodes, thus the interconnections between them are dynamically changing [1]. The absence of infrastructure and the dynamic nature of these networks make them adequate for operating in extreme situations, such as battlefield and disaster recovery [2, 3]. Developing routing protocols for MANETs is highly challenging, due to; continuous variation of the network topology, limited resources in mobile nodes and rapid change of radio propagation conditions [4]. Therefore, recent studies have focused on designing protocols for effective routing, mobility management, data transport, and quality of service (QoS) provisioning [1, 4, 5].

There is a growing requirement to handle real-time applications in MANETs to support their deployment in public domains (e.g., highways, airports, etc.). Numerous routing protocols have been proposed in an attempt to satisfy various objectives, such as efficient utilization of bandwidth and battery capacity [6, 7], optimization of metrics (e.g., throughput and end-to-end delay), fast route convergence, and elimination of loops [8, 9]. QoS routing in *ad hoc* networks is required in many applications [10], yet is challenging to maintain because the network topology may continuously change and the available state information for routing is inherently imprecise [11]. QoS routing may be based on various parameters, such as end-to-end delay, delay jitter, packet loss probability, etc. [9].

Routing problems are classified into unicast and multicast routing. The unicast problem is to find a feasible route from a single source to a single destination. Several routing protocols have been developed to solve the QoS unicast routing problem from different aspects [6–8]. Some works [12, 13] have addressed the problem by offering memetic-based optimization techniques. However, only the delay constraint was considered in these works.

On the other hand, the multicast routing problem aims to find a tree structure, which is used for efficiently delivering the same data stream to different destinations of a network [9]. Different considerations are usually involved when solving the QoS multicast routing problem, such as minimization of routing cost [14], maximization of throughput [15], and minimization of link delay. Therefore, the QoS multicast routing problem is defined as a Non-deterministic Polynomial (NP) hard problem for large-scale and wide area networks [16]. This makes traditional methods of polynomial complexity such as Dijkstra’s algorithm, Bellman-Ford algorithm and Floyd-Warshall algorithm unsuitable for solving this kind of problems for real time applications [12]. Therefore, researchers have studied several metaheuristic methods to solve this problem, such as Ant Colony Optimization (ACO) [17], Particle Swarm Optimization (PSO) [18], Bee Life-based Algorithm (BLA) [19] and Genetic Algorithms (GA) [9, 20, 21]. In those algorithms, first, the fitness of tree structures is analyzed based on the whole network topology, followed by optimization of the tree structures using some meta-heuristic operators.

However, weak robustness, low convergence speed and low search efficiency pose limitations to the performance of those algorithms.

Interesting results from previous works [12, 13] in solving the unicast routing problem using memetic algorithm, encouraged us to investigate the use of memetic algorithm in solving the QoS multicast routing problem which is a non- deterministic polynomial hard problem. In [22], Bäck and Schütz suggested a time-dependent mutation rate to solve difficult combinatorial optimization problems (multiple knapsack and maximum independent set). The performance of their self-adaptation method was significantly better than the performance of other conventional algorithms in terms of convergence reliability and velocity. We have extended their work on GA to develop a new MA that uses an adaptive mutation parameter to solve the multicast routing problem and support QoS-featured routing, based on four different QoS parameters. The performance of the proposed MA is analyzed with regard to three important QoS constraints: the end-to-end delay, the bandwidth and the delay jitter. The QoS constraints and the network cost of the multicast tree constitute the objective function that our algorithm thrives to minimize. To the best of the authors’ knowledge, no previous study has simultaneously analyzed all MA parameters, including population size, maximum iteration number, mutation, and crossover probabilities. The present study proposes a new taxonomy of MA parameters and performs an extensive analysis of these components to determine the best levels for all parameters in the context of MANET routing.

The rest of this paper is organized as follows: Section 2 presents a description of the QoS multicast routing problem. Section 3 explains the development of MA. In Section 4, the algorithm complexity analysis is presented. Section 5 discusses the formulation and testing of the experimental design and demonstrates simulation results. Finally, Section 6 discusses the results obtained and concludes the study.

## QoS multicast routing problem

**Fig 1:**
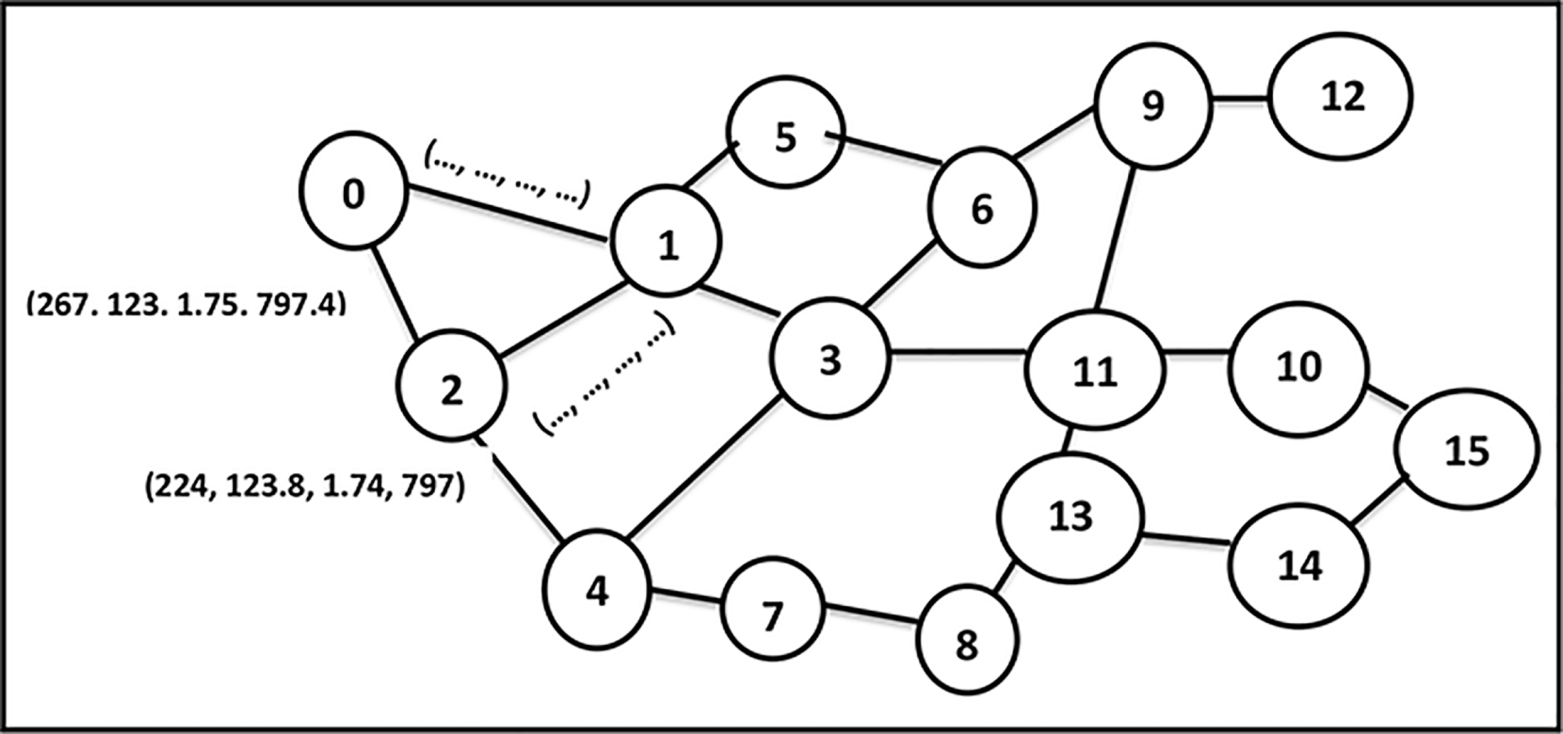
MANET example.

An *ad hoc* network model is presented as an undirected weighted graph *G = (M*, *L)* where *M* denotes the set of nodes labeled as (1, 2, 3, …., *m*), and *L* denotes the set of communication links connecting two neighboring nodes. Each of these communication links indicates the existence of a radio interface for each node, with a common wireless channel, and all of the links have weights representing link transmission cost, delay values, link transmission delay variation (jitter), and estimated link bandwidth. Fig 1 shows an example of an *ad hoc* network consisting of 16 nodes. As an example, the link between node 0 and node 2 has a link cost = 267, delay = 123ms, jitter = 1.75ms and bandwidth = 797.4 Kbit/s.

In order to transfer a data message from the source *s* (where *s ϵ M*) to a group of destinations *D* (where *D ϵ* {*M—s*}), a multicast tree *T(s*, *D)* is generated from the nodes of graph *G*. A multicast tree *T(s*, *D)* represents a group of different paths from node *s* to all destinations *D*. Therefore, the objective of a multicast routing problem is to find a multicast tree *T(s*, *D)* that has a set of paths with acceptable QoS metrics. In the present study, four QoS constraints are considered in solving a multicast routing problem: transmission cost, end-to-end delay, delay jitter, and available bandwidth. According to [19], the total cost of *T(s*, *D)* is given by Cost(*T(s*, *D)*) = ∑_*l∈ T*(*s*,*D*)_ *C*_*l*_, where *C*_*l*_ is the cost of the communication link (*l*). The delay of a route (*R*) from the source node (*s*) to a destination node (*d*) is equal to the sum of the delay values of all links that form this route Delay(*R(s*, *d)*) = ∑_*l ∈ R*(*s*,*d*)_ *D*_*l*_, where *D*_*l*_ is the transmission delay on the communication link (*l*). Similarly, the end-to-end delay variation (jitter) is given by Jitter(*R(s*, *d)*) = ∑_*l ∈ R*(*s*,*d*)_ *J*_*l*_, where *J*_*l*_ is the delay jitter on the communication link (*l*). The bandwidth of *R(s*, *d)* is the minimum link bandwidth in the entire route, so bandwidth *R(s*, *d)*) = min (bandwidth(*l*)), *l* ϵ *R(s*, *d)*.

Therefore, the current QoS multicast routing problem is formulated around four objectives and the fitness function is to identify a multicast tree *T(s*, *D)* that minimizes the weighted combination of cost, delay, jitter, and bandwidth. This can therefore be defined as follows [19]:

$$\text{Minimize}\;\text{F}(\text{T}(\text{s},\;\text{D}))\;=\;w_{1}\text{Cost}(\text{T}(\text{s},\;\text{D}))+w_{2}\text{Delay}(\text{R}(\text{s},\;\text{d}))\;+w_{3}\text{Jitter}(\text{R}(\text{s},\text{d}))+w_{4}\text{bandwidth}(\text{R}(\text{s},\text{d}))$$ (1)

Subject to:

$$\text{Delay}(\text{R}(\text{s},\;\text{d}))\;\leq \;Q_{d}$$ (2)

$$\text{Jitter}(R(s,\;d))\;\leq \;Q_{J}$$ (3)

$$\text{bandwidth}(R(s,\;d))\;\geq \;Q_{b}$$ (4)

Where *w*_1_, *w*_2_, *w*_3_ and *w*_4_ are the objective weighting coefficients used to evaluate the problem based on the relative importance of these four objectives. Furthermore, *Q*_*d*_ is the upper bound of the delay, *Q*_*J*_ is the upper bound of the jitter and *Q*_*b*_ is the lower bound of the bandwidth of every route.

## Adaptive mutation based MA scheme

Although many studies have evaluated the improvement of the QoS of MANET routing problems using genetic algorithms (GA) [1, 5, 9, 20, 21], yet few of them employed MA in their applications. In addition, several studies [23, 24] showed that MA outperforms GA in many applications. Since, MA includes a local search process; it is helpful in effectively solving different optimization problems [23].

Although MA generally appears to be a single robust algorithm, which contains the same operators and holds the same algorithmic logic, the algorithm itself is, in fact, significantly different for fully-defined domains [25]. The main reason behind this is that the Evolutionary Algorithm (EA) has several parameters, and any combination of these parameters has different impact on the performance of the EA [26, 27]. These parameters are classified into two categories: structural and numerical. Structural parameters are the main factors affecting the EA performance, and the most complex set of parameters to be addressed in an EA application [28]. As the name of the category suggests, they are concerned with the structure of an EA, hence any change in a structural parameter value calls for significant alterations in the coding pattern of the EA. The entire algorithm must then be reformulated, including the coding scheme, operator types, and stopping criterion. Numerical parameters constitute the second category of the taxonomy. The initial population, generation number, population size, crossover, and mutation probabilities are the primary variables recognized in this category. These parameters are easy to manage when the coding structure is established. Although modifications in the levels of these parameters do not require extensive coding, a different combination leads to drastic changes in EA performance [29, 30]. Several previous studies have analyzed numerical parameters; however, none made general conclusions regarding computing optimum values for these parameters [31]. Moreover, these studies analyzed the parameters separately, ignoring the interaction between different parameters, which introduces a gap in the literature when it comes to numerical parameters.

DeJong [32] and Fogarty [33] attempted to identify the optimal control parameters for the EA on a chromosomal representation, and concluded that if the mutation rate is too high, then the search is random, irrespective of other parameter settings. They suggested optimal population size values in the range of 50–100, a mutation probability of 0.001, and a single-point crossover with a rate in the range of 0.6–0.95. Grefenstette [34] reported that, in small populations in the range of 20–40, good performance is generally coupled with either a high crossover rate and a low mutation rate, or a low crossover rate and a high mutation rate. [34] also concluded that a mutation rate above 0.05 is generally detrimental to the optimal performance of EAs, when optimal control parameters with a population size of 30 individuals, a mutation rate of 0.01, and a two-point crossover rate of 0.95 are deployed. Schaffer et al. [35] observed that EA performance has a greater sensitivity to mutation rate than to crossover rate, using the same optimal parameter settings, suggested by Grefenstette [34]. In a population size of 20–30 individuals, the optimal mutation rate was established as between 0.005 and 0.01, and the optimal crossover rate in a range of 0.75–0.95. However, Alander [36] reported that an approximation value between n and 2n is optimal for the population size, where n equals the number of network nodes. A typical size of 10–40 population is common in MAs, because the computational complexity of the local search does not permit the evolution of much larger populations in real time applications [23, 24]. As per the results reported in [34–36], the levels of population size are taken as 10, 20, and 40.

Several previous studies [33, 37, 38] showed that mutation probability should be decreased during the convergence stage, so that each chromosome has the crossover probability *p*_*c*_ and the mutation probability *p*_*m*_ necessary to undergo crossover and mutation, respectively. During the execution of EAs, *p*_*m*_ is adapted based on generation progress, with probabilities being large when the mean fitness is near the initial process, and small for particular chromosomes with near optimum finesses. Mühlenbein [39] investigated the optimal mutation rate for a single parent that generates offspring with improved survival and fitness for the next generation. For example, Mühlenbein [39] recommends an optimal approximation of the mutation rate of *p*_*m*_ = 1/n (where n is the number of nodes in network) to improve the objective function value. This setting yields surprisingly good results for a variety of NP-hard combinatorial optimization problems, such as the maximum independent set problem [40], the multiple knapsack problem [41], and the minimum vertex cover problem [42]. Bäck and Schütz [22] suggested a time-dependent mutation rate *p*_*m*_ as follows:

$$p_{m}\;=\;{\left(2+(\frac{n-2}{T-1})t\right)}^{-1}$$ (5)

Where t ∈ {0, 1, ….., T– 1} denotes the generation counter, and T is a given maximum number of generations. From the range of $\;{\;p}_{m}\;=\;\frac{1}{2}–\frac{1}{n}$, the adapted intelligent mutation rate described in Eq (5) can easily satisfy that range. During the execution process, a new mutation rate value is created for each generation within that range, so it is certainly plausible to state that Eq (5) better serves as a general parameter setting rule than does $p_{m}\;=\;\frac{1}{n}$.

The present study examines the effects of MA primary parameters on performance, with regard to the optimal fitness identified for *ad hoc* network routing problems, and proposes guidelines for parameter selection for this particular problem domain. Test problems of the *ad hoc* network, which are extracted from previous studies, are selected for simulation [19]. The present study has two main aims; first, to propose a new taxonomy for MA parameters, and second, to present an extensive analysis of these parameters to draw a general conclusion applicable to an *ad hoc* network domain.

**Fig 2:**
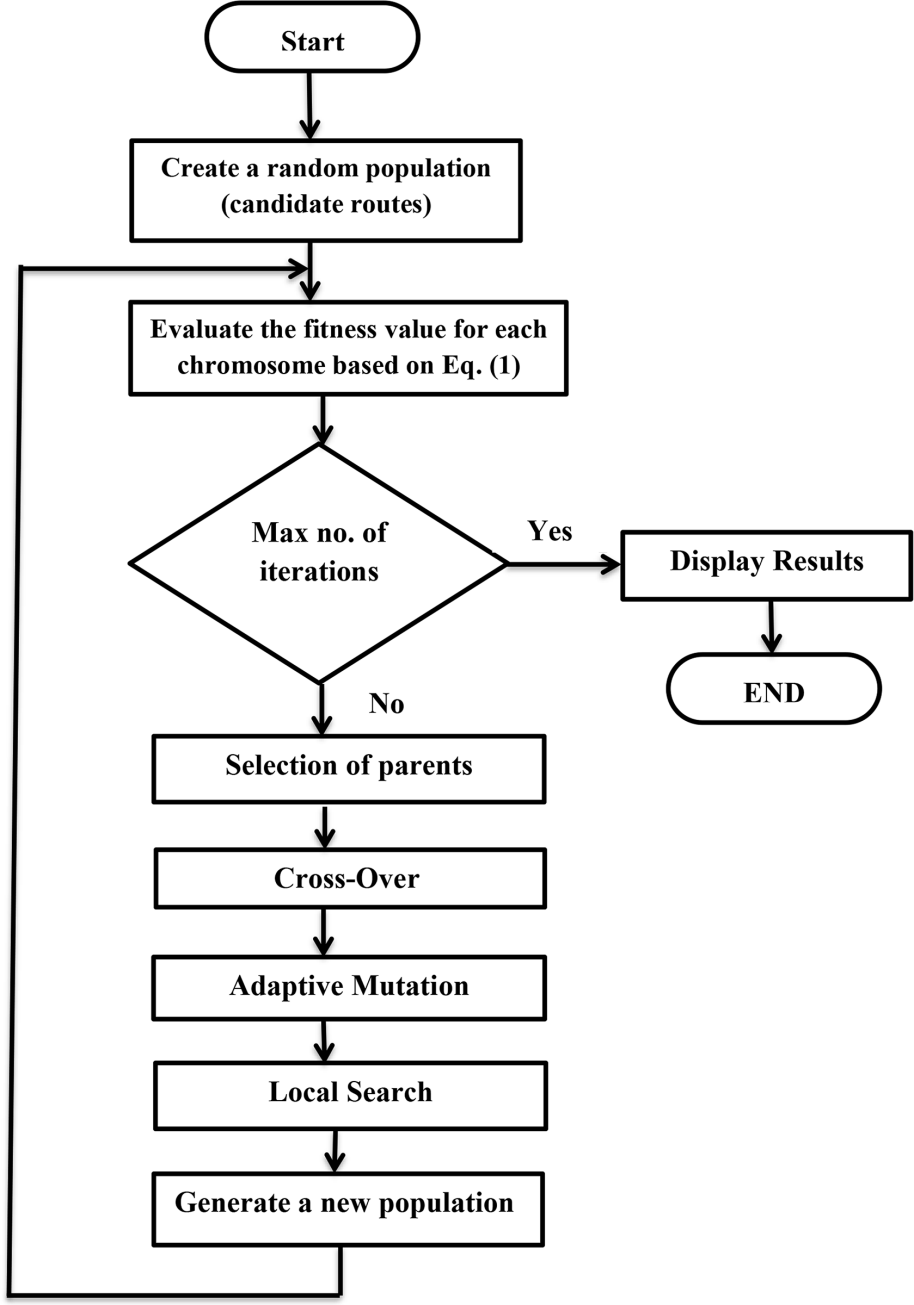
Memetic algorithm flow chart.

An MA provides a near-optimal solution by evolving a group of successive generations. As shown in Fig 2, the first generation is randomly selected from the total population pool. In addition, the total population should be very high so that it is not possible to computationally evaluate every solution in the whole pool. Otherwise, if the population is not large enough then evaluating each solution separately and selecting the best would be more efficient than using the memetic algorithm.

Parent selection is made by tournament selection, where two individuals are randomly selected, and one-point crossover is carried out. The crossover point is selected randomly, but only at the valid boundaries, since the main concept of crossover is to pass on the genetic information [43]. After completion of the crossover operation, the two new offspring are evaluated. If the fitness of a single offspring is superior to the fitness of either parent, the new offspring is selected. This operation is carried out to reflect the contribution of the genes to the improvement in fitness. The proposed MA is explained in the following Pseudocode.

**PseudoCode 1:** Proposed MA

1. Create a random population of n = 40 multicast trees (chromosomes).
2. Evaluate the fitness function *F(i)* of each chromosome (*i)* in the population based on Eq (1).
3. While (end condition not satisfied) do {
  - Perform crossover for the selected parents with probability (*p*_*c*_ = 0.85), to form new offspring.
  - Execute mutation on selected chromosomes with probability described in Eq (5).
  - Conduct a local search procedure (a mixture of greedy and steepest approaches) for selected chromosomes.
  - Place the new accepted offspring (feasible solutions) to form a new population.
  - [Replace old population] Use the newly generated population for a further run of the algorithm.
  - [Test] If the end condition is satisfied (iteration number was set to 50), stop, and display the optimal routes in the current population.
  - }

A chromosome (single solution) is a multicast tree that contains the different routes from source node to each one of the multicast group members (destinations) via a set of intermediate nodes. A route is represented as a string of nodes that link the source to one of the destinations. Then, the population is a set of multicast trees (feasible solutions).

Finally, the local search implemented is a mixture of greedy and steepest approaches. First, the procedure begins with the random selection of one gene (node) removal position *i* (the greedy approach). The location of this gene is then removed. Second, the local search picks an insertion position *j* for the removed location. The optimal insertion position is then selected from all of those possible (the steepest approach). If the gene insertion at the optimal position results in an overall improvement of the objective function (fitness value), accounting for both changes due to the removal and the insertion, the shift move is performed; otherwise, the process is switched back.

## Complexity analysis of the proposed scheme

In order to describe the computational resource running time of the MA, the complexity is computed as follows. Let N and M be the number of nodes in the network, and the number of chromosomes (solutions), respectively. The MA starts off using O(M × (n − 1) × log(n − 1)) time units to develop randomly M chromosomes. The MA iterates by letting *p*_*c*_ be the crossover probability and *p*_*m*_ be the value of the mutation probability. The number of offspring developed by crossover consumes O(N × *p*_*c*_ × [M × (N + 1)]) time units, while the number of mutated offspring consumes O(*p*_*m*_ × [M × (N + 1)]) time units. Since adaptive mutation rate described in Eq (5) is used, we consider $p_{m}\;=\;\frac{1}{2}$ as the upper bound value. This concerns the reproduction phase. Finally, a local search is introduced which spends {O(N) + N × O(1)} ≈ O(N) time units. Thus, the total time complexity for each iteration is recorded as:: O(M × (n − 1) × log(n − 1)) + O(N × *pc* × [M × (N + 1)]) + O(1/2 × [M × (N + 1)]) + O(N). The total computational complexity of the MA is represented in Eq (6).

O((M×(n-1)×log(n-1))+(N×pc×[M×(N+1)])+(1/2×[M×(N+1)]+N)) (6)

From Eq (6), it is clear that the MA has a linear complexity that is reasonable in time and in developing the lowest-cost network trees. In addition, the population size of MAs is typically smaller when compared to GAs [24]. This small population size allows the MA to have a faster convergence rate.

Computational complexities of EAs with different coding schemes and operators are hardly described uniformly. Hence, we just focus on the computational complexities of crossover operators. As in [9, 19], the complexity of BLA at the cross over stage for (*p*_*c*_ = 0.85) is in the order of (1.5 × M), while that of ISGSA and EEGA is of order (M^2^). However, in the proposed MA algorithm, the cross over stage requires a complexity in the order of (0.85 × M).

## Proposed algorithm simulation

The main aim of the proposed MA is to solve the multicast routing problem by finding a multicast tree of paths from source to different destinations that offers optimal fitness value. This optimal fitness is the minimized value of the weighted combination between the optimization metrics (cost, delay, jitter and bandwidth) calculated by Eq (1). All experiments were implemented on MATLAB 2016a, with an Intel ^®^ Core ^™^ i5-4200M CPU, and 6 GB RAM.

### A. Simulation parameters setup

A test problem from a previous study is used [9] to determine the optimal MA parameters, based on the simulation-optimization technique, and the significance of different values of these parameters using factorial design and Analysis Of VAriance (ANOVA). The optimal fitness value obtained is the performance measure considered throughout the analysis. The objective function value (the fitness value), one-point crossover with a probability of *p*_*c*_ (using roulette wheel selection), self-adaptive mutation with a probability of *p*_*m*_, and maximum rotation (iteration) numbers as a stopping criterion, were employed in the experimental analysis. The roulette wheel selection procedure was also used for the reproduction process. If unfeasible solutions are reached after the operations of both crossover and mutation, then these chromosomes are discarded, and new ones are generated from the beginning. The system under consideration is an *ad-hoc* network consisting of three separate network models with varying node content (15, 20, and 50 nodes, respectively).

The performance of MA is highly dependent on the initially generated population. Therefore, it is prejudicial to state that one memetic parameter is better than another by simply comparing two solutions with different initialization. To neutralize the effect of the initial population, 10 trials were conducted for each condition in the present study with randomly selected, initial populations. All of the memetic parameters for each evaluation are given in Table 1 except for the mutation rate probability, which is set on according to Eq (5).

**Table 1:** The proposed algorithm settings.

Parameters	Values
Number of generations for a trial (e.g., iterations)	20 and 50
Number of individuals (e.g., chromosomes per generation) [Population size]	10, 20, and 40
Crossover rates	0.6, 0.75, 0.85, and 0.95
Mutation rate	The adapted mutation rate described in Eq (5)
Number of genes (nodes) in each individual (e.g., three different problem sets)	15, 20, and 50

Different static crossover rates are used to examine the variety of results, as shown in Table 1, in order to analyze the impact of variation of crossover rate and to identify the value of the crossover rate that yields the best solution. In addition, an adaptive mutation is used, such that the mutation rate is high in early generations and decreases as generations increase. The selected, adaptive mutation rate is a function of the generation number, and is obtained by Eq (5), where *p*_*m*_ refers to the mutation probability and *n* refers to the number of generations. Since there are three distinct numerical parameters, a three-way factorial design is employed. For each parameter level, 10 replication results, gathered from running the simulation with the assigned parameters, are obtained. ANOVA is used to determine the significance of each effect on the fitness value, using Statistical Package for the Social Sciences Software, version 22 (http://www.spss.com). For each initialized population size, a random search heuristic of 10, 20, and 40 random populations are drawn from all populations. Factors identified as significant are further analyzed using Duncan’s test to differentiate between the factor levels. In addition, if a higher interactive effect is found, a simple effect technique [44] is used to demonstrate the type of effect at each factor level. The dependent variable is the fitness value of the problem and the independent variables are the number of rotations, population size, and crossover rate. The Shapiro and Wilk [45] test is implemented to test the normality of the data, and statistical significance is set at a confidence level of 95%.

After examining data normality, tests for significance are carried out to compare the three assigned parameter levels, based on the evaluation parameters in Table 2a, 2b and 2c. For the 15 nodes network, only population size is significant, as shown in Table 2. Using the Duncan test with a 5% significance level to differentiate between the population size levels, the fitness of the 40 generations (i.e., with a mean value and a standard deviation of 18.675±2.385) was superior to the fitness of both the 20 and 10 generations (i.e., with a mean value and a standard deviation of 24.713±4.401 and 20.725±4.195, respectively).

**Table 2:** Analysis of variance for the 15 nodes network.

Source	Type III Sum of Squares	df	Mean Square	F	Sig.
Rotation	22.204	1	22.204	1.473	0.226
**Pop_Size**	**1508.108**	**2**	**754.054**	**50.031**	**0.000**
Cross_over	7.946	3	2.649	0.176	0.913
Rot*Pop_Size	34.058	2	17.029	1.130	0.325
Rot*Cross_over	9.079	3	3.026	0.201	0.896
Pop_Size * Cross_over	30.592	6	5.099	0.338	0.916
Rot * Pop_Size * Cross_over	8.508	6	1.418	0.094	0.997
Error	3255.500	216	15.072		

Population size is also a significant factor for the 20 nodes network, as shown in Table 3. The fitness of both the 40 and 20 generations (with mean values and standard deviations of 246.464±0.0 and 246.464±0.0, respectively) are superior to the fitness of the 10 generations (254.16±29.993).

**Table 3:** Analysis of variance for the 20 nodes network.

Source	Type III Sum of Squares	df	Mean Square	F	Sig.
Rotation	568.550	1	568.550	1.884	0.171
**Pop_Size**	**3158.910**	**2**	**1579.455**	**5.233**	**0.006**
Cross_over	694.752	3	231.584	0.767	0.513
Rot* Pop_Size	1137.099	2	568.550	1.884	0.154
Rot*Cross_over	695.015	3	231.672	0.768	0.513
Pop_Size * Cross_over	1389.504	6	231.584	0.767	0.596
Rot * Pop_Size * Cross_over	1390.029	6	231.672	0.768	0.596
Error	65190.196	216	301.806		

Population size is also a significant factor for the 50 nodes network, as shown in Table 4. The fitness of the 40 generations (with a mean value and standard deviation of 2.457±0.109) is superior to the fitness of the 20 generations (2.619±0.166), and the fitness of the 20 generations is superior to the fitness of the 10 generations (2.846±0.277). In addition, rotation by crossover interaction is significant; the simple effect technique [44] is employed to describe this interaction. The analysis revealed that the fitness of 50 rotations with a crossover rate of 0.85 (with a mean value and standard deviation of 2.606 ±0.035) is superior to the fitness of 20 rotations with a crossover rate of 0.85 (with a mean value and standard deviation of 2.728±0.035) using t(29) = 2.453, p<0.02. The other comparisons are not significant at the p<0.05 level.

**Table 4:** Analysis of variance for the 50 nodes network.

Source	Type III Sum of Squares	Df	Mean Square	F	Sig.
Rotation	0.000	1	0.000	0.005	0.944
**Pop_Size**	**6.125**	**2**	**3.063**	**82.707**	**0.000**
Cross_over	0.216	3	0.072	1.942	0.124
Rot*Pop_Size	0.024	2	0.012	0.330	0.719
Rotation * Cross_over	**0.317**	**3**	**0.106**	**2.854**	**0.038**
Pop_Size * Cross_over	0.368	6	0.061	1.657	0.133
Rot*Pop_Size * Cross_over	0.243	6	0.040	1.093	0.367
Error	7.998	216	0.037		

On the basis of the statistical results described, it is concluded that the following parameters give the best fitness value for *ad hoc* network domains: 50 rotations, population size of 40 chromosomes, adaptive mutation rate, and 0.85 crossover rate, irrespective of the of the *ad hoc* network size (e.g., number of nodes included).

### B. Simulation results

The efficacy and the validity of the proposed MA are tested via comparing it to other EA techniques. These techniques are; the bee life-based algorithm (BLA) [19], the bees algorithm (BA) [46], the marriage in honey bees optimization algorithm (MBO) [47], the genetic algorithm (GAMRA) [20], the energy efficient genetic algorithm (EEGA) [48], the genetic simulated annealing algorithm (ISGSA) [21], and their updated versions, PM-GAMRA, PM-EEGA, and PM-ISGSA [9]. The simulation is conducted using two datasets. These datasets are available in the Appendix of the study conducted by Liang et al. [9].

The first dataset is a 15-node random graph, which is constructed on the basis of Lu and Zhu [48], where the costs of links are generated in the range between 2 and 39, and delay values are between 1 and 7. Node 0 represents the source node s, and nodes {2, 5, 6, 8, 14} represents the multicast group members (destination nodes). A chromosome is chosen as a multicast tree that contains five paths, with each one being a string of nodes from the source s (node = 0) to one of each of the destination nodes (nodes 2,5,6,8, and 14). The second dataset is 20 nodes, and is generated using a network simulator (http://www.isi.edu/nsnam/ns/), developed by Salim et al. [19]. Node 0 represents the source node s, and nodes {4, 9, 14, 19} represents the multicast group members (destination nodes). A chromosome is chosen as a multicast tree that contains four paths, with each one being a string of nodes from the source s (node = 0) to each of the destination nodes (nodes 4, 9, 14, and 19). In order to evaluate the fitness of each individual (tree structure), Eq (1) is applied, in which the QoS weights are; w_1_ = 10, w_2_ = 1, w_3_ = 10, and w_4_ = 1. The weight values reflect the importance assigned to each one of the four considered QoS parameters [19].

Several measures are introduced to compare the effectiveness and performance of the proposed MA against the aforementioned techniques. These measures are; *R*_*min*_, *R*_*mean*_, and *R*_*variance*_, denoting the minimum, mean, and variance of the results, respectively. The results are all based on K times repeated trials. *R*_*min*_ is computed as min {*R*_*i*, *steps(j)*_, *i = 1*, *2*, …, *K*}, where *R*_*i*, *steps(j)*_ is the optimal solution to the routing problem in the *j* step for the *ith* time computation up to *K*. A similar procedure is followed to compute the *R*_*mean*_ and *R*_*variance*_ measures.

**Fig 3:**
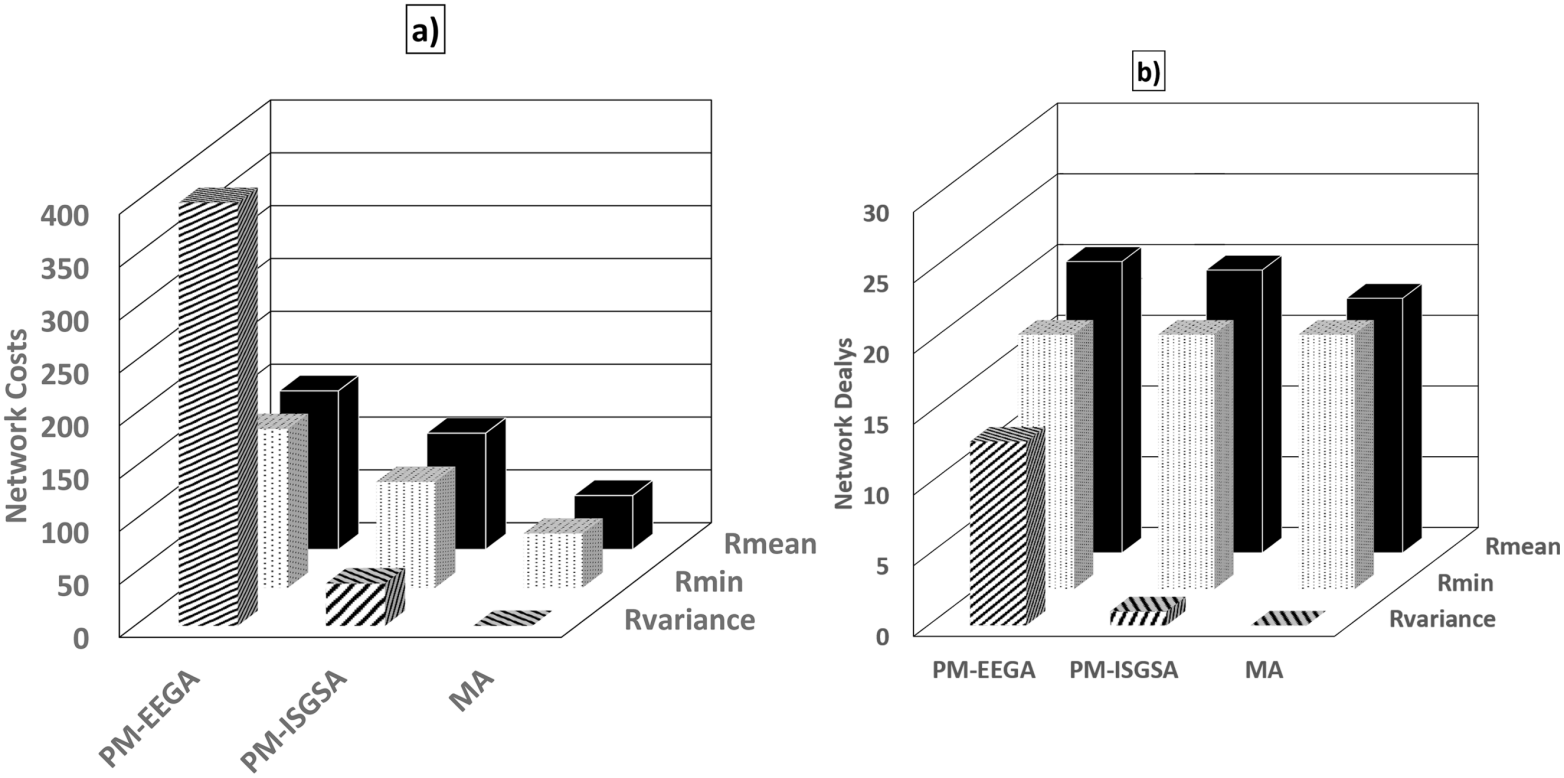
Figs. 3a. and 3b. show the cost and delay comparisons in terms of the mean, min, and variance of the PM-EEGA and PM_ISGSA GAs and MA on the first dataset.

Fig 3 shows the *R*_*min*_, *R*_*mean*_, and *R*_*variance*_ of the results obtained via PM-GAMRA, PM-ISGSA [9], and the proposed MA, using the first dataset. It is clear from the figure that the proposed MA provides mean cost = 86 and mean delay = 18. On the other hand, the former two GAs provide mean cost = 108 and mean delay = 18. Moreover, the *R*_*variance*_ of the proposed MA is zero value which is less than those reported for the former GAs, which indicates that the proposed MA is more robust.

Both PM-EEGA and PM-ISGSA generate near optimum solutions in the evolution, yet their costs and fitness results, did not reach the level of success of the proposed MA results. Fig 3a and 3b show that the *R*_*min*_, *R*_*mean*_, and *R*_*variance*_ of the MA fitness value are clearly less than those of PM-EEGA and PM-ISGSA. These results indicate that the MA scheme improved searchability as to the optimal solution.

**Fig 4:**
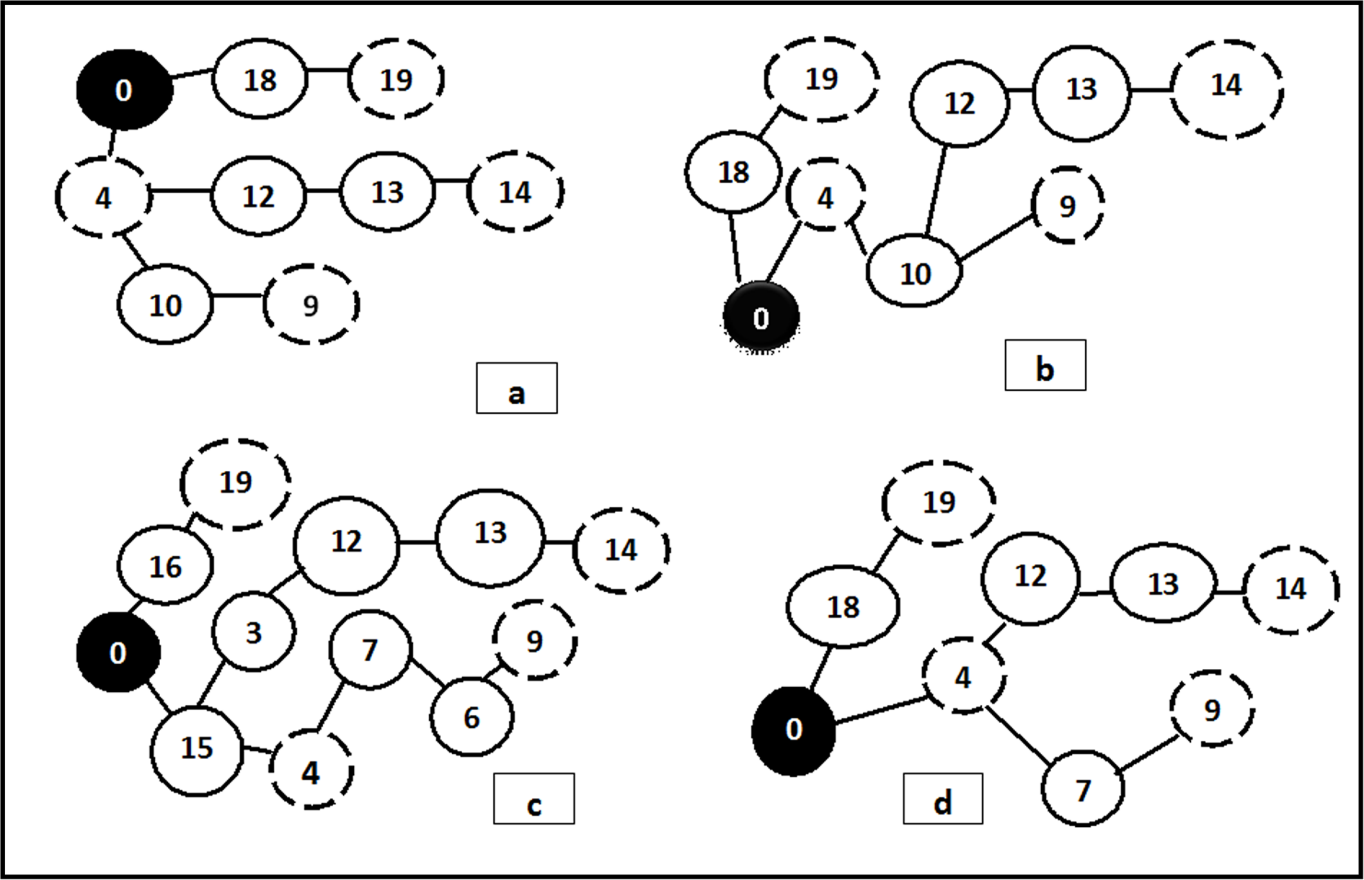
The optimal multicast trees for: (a) MA, (b) BLA, (c) BA, and (d) MBO for the second dataset.

Fig 4 shows the optimal multicast trees reached by the proposed MA, BLA, BA, and MBO, respectively, using the second dataset. The MA fitness result is compared in terms of common QoS constraints (cost, delay, jitter, and bandwidth) with the other meta-heuristic algorithms; MBO [47], BA [46], BLA [19], PM-EEGA, and PM-ISGSA [9]. The fitness values of these algorithms (as listed in Table 5) are induced from [9].

**Table 5:** The fitness and property values of the optimal results generated by the different algorithms on the applied dataset. The bold numbers emphasize the optimal values of properties.

Algorithms	MA	BLA	BA	MBO	PM-EEGA	PM-ISGSA
**Fitness**	**18822.5**	18905.29	20266.64	18987.34	18905.29	18822.64
**Cost**	1746.19	**1740.38**	1876.53	1762.65	**1740.38**	1746.29
**Delay**	**493.14**	616.34	616.13	**493.14**	616.34	**493.14**
**Jitter**	**7**	8.75	8.75	**7**	8.75	**7**
**Bandwidth**	797.5	797.54	797.65	797.57	797.54	797.54

**Fig 5:**
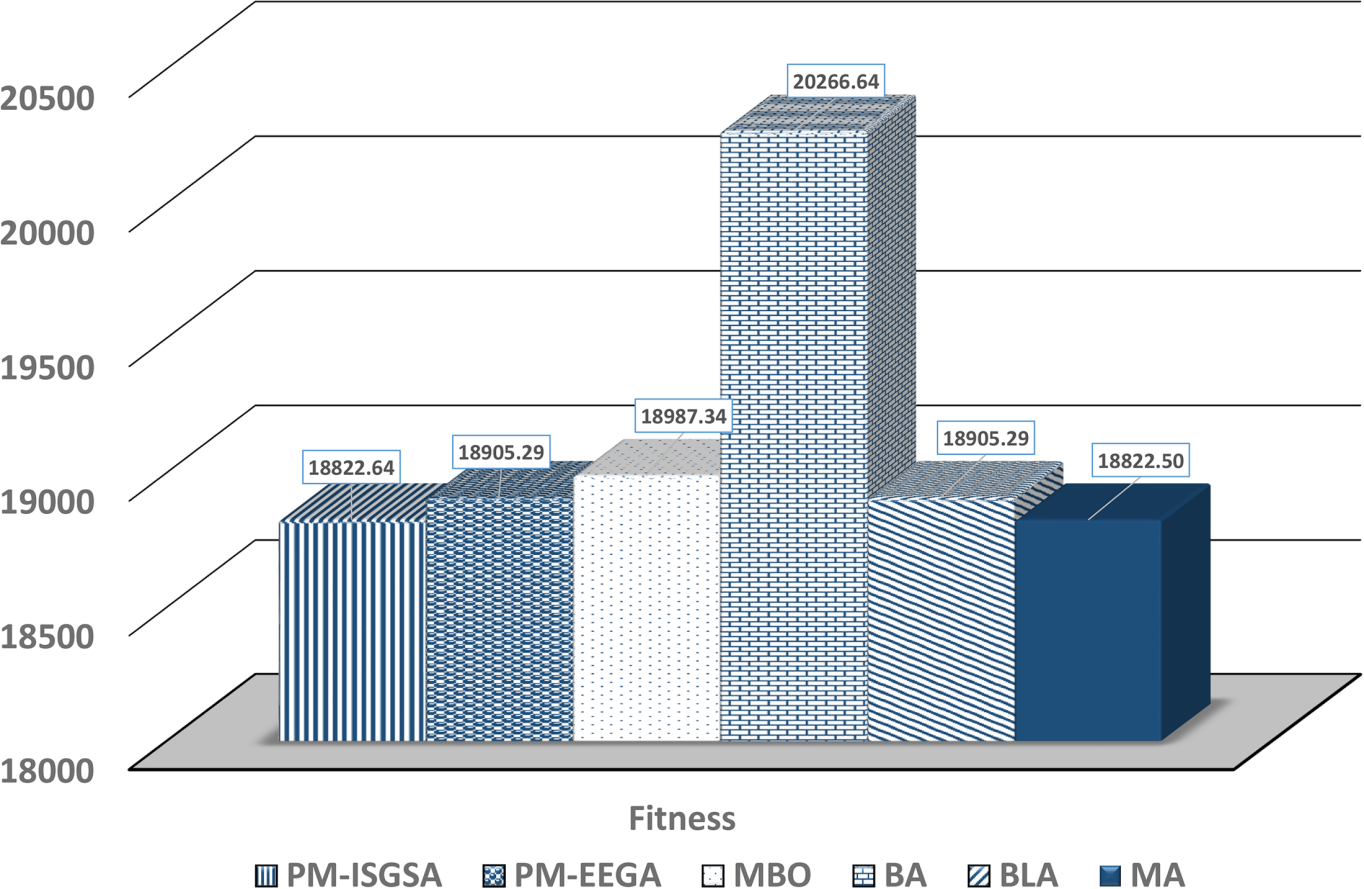
The optimal fitness solutions identified by applying different algorithms on the second dataset.

The results in Fig 5 show that the fitness values of the proposed MA scheme are lower than those of the other algorithms. This indicates that the proposed MA scheme can offer a high level of robustness for *ad hoc* networks by constantly identifying optimal solutions, supported by a 72% success rate.

**Fig 6:**
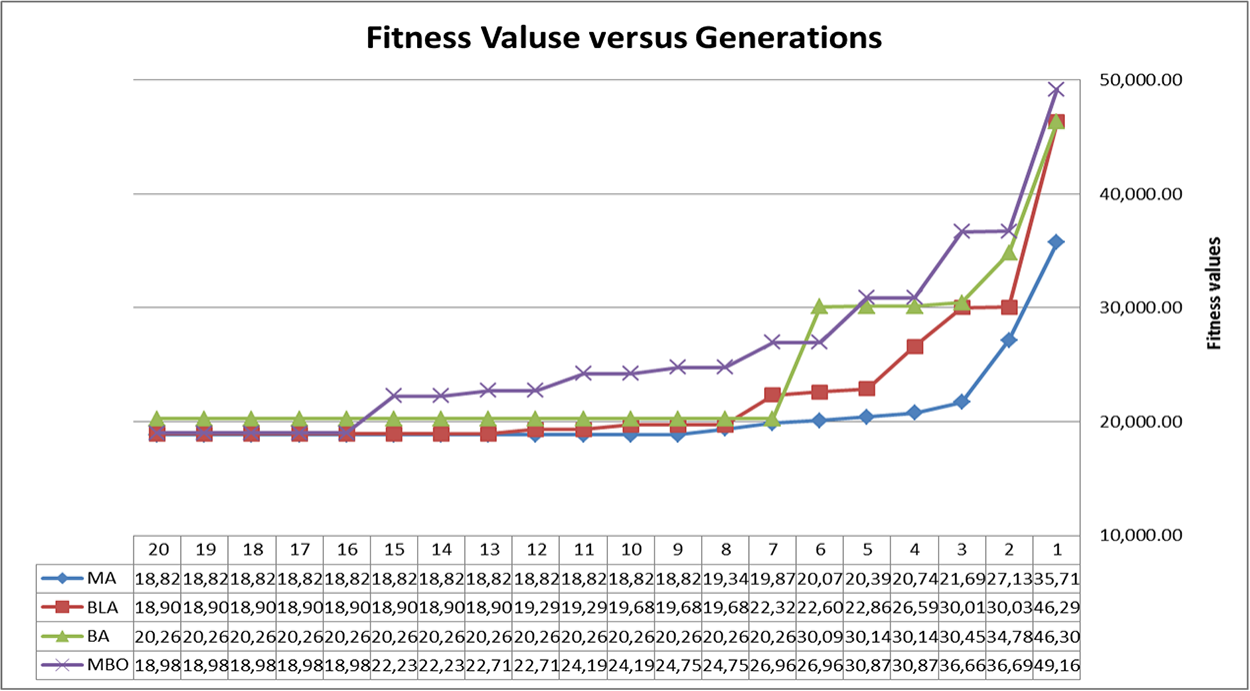
The optimal fitness solution in generations (iteration times).

In order to compare the execution time of MA against other approaches (BLA, BA, and MBO), the improvement progress for the algorithms is summarized in Fig 6 and Table 6. This figure illustrates the stagnation of the optimal fitness solution for each generation (iteration times) developed by each algorithm. It is observed that the proposed MA algorithm reaches the optimal fitness (18,822.5) in a reasonable number of generations (the 9th generation). This shows that a reasonable convergence speed is associated with the low fitness solution compared to other approaches. For example, the MBO algorithm reaches its stagnation state at the 16th generation, with a fitness value of 18987.34. However, BA algorithm reaches its stagnation state at the 7th generation, but with a poor fitness value (20,266.64). In this case, BA dropped early in local optimal value. Also, BLA reached optimal fitness (18,905.29) in the 13^th^ generation.

**Table 6:** A comparison of optimal fitness solutions in generations (iteration times).

Generation	MA	BLA	BA	MBO
1	35,717.64	46,290.62	46,309.36	49,163.7
2	27,134.38	30,032.38	34,786.42	36,696.46
3	21,692.767	30,015.78	30,457.64	36,667.44
4	20,749.467	26,591.38	30,143.22	30,875.04
5	20,390.946	22,862.6	30,143.22	30,875.04
6	20,075.922	22,606.02	30,093.62	26,969.8
7	19,874.169	22,324.82	**20,266.64**	26,969.8
8	19,340.803	19,683.8	20,266.64	24,757.2
9	**18,822.5**	19,683.8	20,266.64	24,757.2
10	18,822.5	19,683.8	20,266.64	24,197
11	18,822.5	19,297	20,266.64	24,197
12	18,822.5	19,297	20,266.64	22,717
13	18,822.5	**18,905.29**	20,266.64	22,717
14	18,822.5	18,905.29	20,266.64	22,237
15	18,822.5	18,905.29	20,266.64	22,237
16	18,822.5	18,905.29	20,266.64	**18,987.34**
17	18,822.5	18,905.29	20,266.64	18,987.34
^^^^^^^^^^	^^^^^^^^^^	^^^^^^^^^^	^^^^^^^^^^	^^^^^^^^^^
50	18,822.5	18,905.29	20,266.64	18,987.34

To sum up, the above results showed the effectiveness of the proposed adaptive mutation-based memetic algorithm (MA) which combines the advantages of both genetic algorithm and local search method. Combining both techniques in solving the multi-cast routing problem outperformed MBO [47], BA [46], BLA [19], and the PM-modified Physarum network model [9]. The use of local search technique allows the algorithm to avoid premature convergence to suboptimal solutions. According to the obtained results, the best fitness is the fitness obtained by MA (18,822.5), achieving lowest delay (493.14ms) and lowest jitter (7ms) with a reasonable transmission cost value. Other algorithms that achieved the same delay or jitter performance, obtained similar performance at a higher transmission cost. As a result, the proposed algorithm attained the minimum overall fitness value. Even though the performance of proposed MA and PM-ISGSA is very close, the variance of all parameters is much lower for MA, which leads to a more robust and stable performance. In addition, due to the fact that the proposed MA parameters were optimized based on statistical analysis, the proposed MA is more efficient compared to other algorithms in terms of convergence speed and computational complexity.

## Discussion and conclusion

This paper proposes a new adaptive mutation based MA to solve the quality of service multicast routing problem for *ad hoc* networks. The proposed MA uses an adaptive mutation parameter. Four metrics were used to test the reliability and the efficiency of the new algorithm: transmission cost, delay, jitter and bandwidth.

The results of the performed statistical analysis revealed that 50 rotations, 40 chromosomes population, adaptive mutation rate, and 0.85 crossover rate provided the optimal fit and were sufficient for *ad-hoc* network applications. These findings are in accordance with the recommendations of previous studies [34–36, 39, 22].

Through simulation, the proposed MA was tested using the dataset published in the study conducted by Liang et al. [9]. The proposed MA demonstrated promising results in terms of its fitness values when compared to the MBO [47], BA [46], BLA [19], and the PM-modified Physarum network model [9]. Remarkably, MA outperformed other algorithms by achieving minimum fitness value = 18,822.5, lowest delay = 493.14ms, and lowest jitter = 7ms. The proposed MA provided the minimum value for most of the tested runs (i.e., supported by a success rate of 72%). The reasons behind this success rate are; the local search included in the MA and an adaptive mutation component, in which mutation probability decreased during convergence stage.

To sum up, the numerical results obtained from previous studies were utilized to test the proposed adaptive mutation parameter that was built in the MA, using a range of commonly used changeable functions. The results of these examinations demonstrated that the form of adaptive MA is capable of creating more robust search performances than its authoritative evolutionary counterparts.

## Supporting information

S1 File Data set. (DOCX)
