# Supplementary material for: A memetic optimization algorithm for multi-constrained multicast routing in ad hoc networks
Source: PLoS One. 2018 Mar 6;13(3):e0193142. doi: 10.1371/journal.pone.0193142 (PMC5839550; doi:10.1371/journal.pone.0193142)
Supplement: S1 File — (DOCX) [file pone.0193142.s001.docx]

First Dataset:

D_1_ = {(number of endpoint, number of endpoint, cost, delay)| (2, 1, 11, 4) (3, 1, 7, 6) (8, 1, 21, 3) (4, 2, 4, 4) (5, 2, 2, 5) (11, 2, 5, 3) (4, 3, 24, 3) (6, 4, 7, 11) (6, 5, 39, 1) (12, 5, 3, 5) (7, 6, 22, 3) (14, 6, 35, 4) (12, 7, 15, 4) (13, 7, 2, 3) (9, 8, 21, 3) (10, 8, 14, 6) (10, 9, 10, 7) (12, 9, 13, 1) (12, 10, 20, 3) (12, 11, 21, 3) (15, 13, 5, 3) (15, 14, 6, 4)}

Second Dataset:

D_2_ = {(number of endpoint, number of endpoint, cost, delay, jitter, bandwidth)| (1, 2, 229.2, 123.27, 1.751,797.457) (1, 5, 277.884, 123.312, 1.7516, 797.403) (1, 16,153.24, 123.205, 1.75, 797.539) (1, 17, 132.163, 123.187,1.7497, 797.562) (1, 18, 53.8145, 123.12, 1.7487, 797.648)(1, 19, 168.808, 123.219, 1.7502, 797.522) (2, 3, 215.969,123.259, 1.7508, 797.471) (2, 4, 267.247, 123.303, 1.7515,797.415) (2, 5, 63.7104, 123.129, 1.7488, 797.637) (2, 6,176.894, 123.226, 1.7503, 797.513) (2, 8, 152.6, 123.205,1.75, 797.54) (2, 9, 239.438, 123.279, 1.7511, 797.446) (2,11, 202.107, 123.247, 1.7506, 797.486) (2, 12, 266.533,123.302, 1.7515, 797.416) (2, 16, 126.895, 123.183,1.7496, 797.568) (2, 18, 197.297, 123.243, 1.7506,797.491) (3, 5, 260.038, 123.297, 1.7514, 797.423) (3, 6,63.0259, 123.128, 1.7488, 797.638) (3, 8, 248.496, 123.287, 1.7513, 797.435) (3, 9, 80, 123.143, 1.749, 797.62) (4, 5, 221.549, 123.264, 1.7509, 797.465) (4, 8, 294.157, 123.326, 1.7519, 797.386) (4, 11, 244.492, 123.283, 1.7512, 797.44) (4, 12, 82.8908, 123.145, 1.7491, 797.616) (4, 13, 52.5624, 123.119, 1.7487, 797.65) (4, 16,224.185, 123.266, 1.7509, 797.462) (5, 6, 210.481, 123.254, 1.7508, 797.477) (5, 8, 116.669, 123.174, 1.7495, 797.579) (5, 9, 266.66, 123.302, 1.7515, 797.416) (5, 11, 144.867, 123.198, 1.7499, 797.549) (5, 12, 208.471, 123.253, 1.7507, 797.479) (5, 13, 255.831, 123.293, 1.7514, 797.427) (5, 16, 147.683, 123.2, 1.7499, 797.545) (5, 18, 238.255, 123.278, 1.7511, 797.447) (6, 8, 186.141, 123.233, 1.7504, 797.504) (6, 9, 64.9019, 123.13, 1.7488, 797.636) (6, 11, 272.403, 123.307, 1.7516, 797.409) (6, 16, 294.214, 123.326, 1.7519, 797.386) (7, 8, 239.9, 123.279, 1.7511, 797.445) (7, 10, 84.9586, 123.147, 1.7491, 797.614) (7, 11, 221.828, 123.264, 1.7509, 797.464) (8, 9, 219.14, 123.262, 1.7509, 797.468) (8, 10, 280.165, 123.314, 1.7517, 797.401) (8, 11, 86.539, 123.148, 1.7491, 797.612) (8, 12, 248.458, 123.287, 1.7513, 797.435) (8, 16, 263.536, 123.3, 1.7515, 797.419) (10, 11, 235.5, 123.276, 1.7511, 797.449) (11, 12, 182.129, 123.23, 1.7504, 797.508) (11, 13, 250.027, 123.288, 1.7513, 797.434) (11, 16, 284.1, 123.317, 1.7517, 797.397) (12, 13, 68.656, 123.133, 1.7489, 797.632) (12, 16, 262.887, 123.299, 1.7515, 797.419) (13, 14, 264.3, 123.3, 1.7515, 797.418) (13, 16, 275.18, 123.31, 1.7516, 797.406) (14, 15, 199, 123.244, 1.7506, 797.489) (16, 17, 235.9, 123.276, 1.7511, 797.449) (16, 18, 102.801, 123.162, 1.7493, 797.595) (16, 19, 297.402, 123.329, 1.7519, 797.382) (17, 18, 146.756, 123.2, 1.7499, 797.547) (17, 19, 71.1157, 123.135, 1.7489, 797.629) (17, 20, 261.873, 123.298, 1.7514, 797.421) (18, 19, 200, 123.245, 1.7506, 797.489) (19, 20, 200, 123.245, 1.7506, 797.489)}
